# Contemporary Integrative Review in Simulation-Based Learning in Nursing

**DOI:** 10.3390/ijerph18020726

**Published:** 2021-01-15

**Authors:** Vasuki Rajaguru, Jungmin Park

**Affiliations:** 1 College of Medicine, Ajou University, Suwon 16499, Korea; vasuki81@daum.net; 2 School of Nursing, Hanyang University, Seoul 04763, Korea

**Keywords:** simulation, learning, literature review, nurse education, nursing students

## Abstract

*Background:* In general, simulation-based learning (SBL) has been a part of nursing education in the past two decades, though nursing educators are facing difficulties in evaluating its effectiveness in theory and practice. The aim of this review was to synthesize the research findings regarding the effects of SBL among nursing students from published scientific articles. *Methods:* This integrative review focused on articles published in English between 2016 and 2020, identified by using electronic databases such as Cochrane Library, PubMed, Medline (Ovid), SCOPUS, RISS, and Korea Med. Results: Fifteen articles were selected by a step-by-step process. Data were synthesized and effects of SBL methods were derived under four themes: ‘Knows A Self Com_p’ such as knowledge and skills; Attitude; Self (learning, efficacy, determination, competency, confidence, utilization, satisfaction, assessment); and Com(*n*) (competency, communication, and confidence) and P (perceptions and performance). *Conclusion:* The review results showed that SBL is the best method for teaching clinical practice. Article-focused simulations with simulators were more effective than classroom teaching, depending on the context, topic, and method. The overall results showed that SBL is a positive, safe and effective method for nursing students in clinical and simulation room settings to improve the skills and practice of client care.

## 1. Introduction

Nursing education consists of two-way methods that provide theoretical and practical training to nursing students with the purpose of preparing them for their duties in nursing care. Nursing educators have been facing challenges in integrating theory and practice for a long time; often, this is referred to as a theory and practice gap [1]. One important outcome of nursing education is assessing, planning, implementing, and evaluating patients’ needs. The Future of Nursing, reported by International Organization for Migration [2], states that changes in advancing health education provide a framework for nurse educators to incorporate simulation and promote integrated learning experiences. These cover all basic nursing skill requirements that students should begin to develop during their study period. In skill-based education, where learning by practice plays an important role, it is essential to ensure the integration of theoretical knowledge with practice [3]. Therefore, simulation-based learning (SBL) was introduced. This paper outlines how students learn from SBL, followed by a presentation and debriefing.

Simulation is increasingly used as an effective learning method, especially in the area of healthcare [4]. Simulation is a type of reality-based education for certain tasks, relationships, phenomena, equipment, behaviors, and cognitive activities that occur in everyday clinical practice [5]. Notably, the International Nursing Association for Clinical Simulation and Learning (INASCL) explained that, “Simulation-based experiences are purposefully designed to meet identified objectives and optimize achievement of expected outcomes [6]”.

Simulation-based learning integrates cognitive, technical, and behavioral skills into an environment where learners believe the setting is real, act as they would respond in the field, and feel safe to make mistakes for the purpose of learning from them [7]. SBL has been explored by the nursing professional bodies and nursing educators [8,9].

However, due to recent changes in nursing education, nursing faculty are subjected to heavy workloads. Although they are committed to learning about simulation-based technology, isolation within one’s area of expertise, or the ‘‘silo’’ effect, along with a lack of experience in the development of instrumentation and research may be reasons for the delay in introducing vetted and effective SBL experiences throughout nursing curricula. Determining the current state of knowledge regarding the evaluation of simulation in nursing is necessary for educators to improve research methods, educational efforts, and student outcomes.

The purpose of this study was to examine the effects of SBL in nursing education by synthesizing articles based on their measurement methods.

## 2. Methods

### 2.1. Integrative Review

This integrative review focused Whittemore and Knafl [10] methodology by using the following steps: literature or data search, data evaluation/reduction, data comparison/synthesis, and presentation. The method is further enhanced the by use of themes to interpret the broad set of identified articles within the review.

### 2.2. Data Search

This integrative review included articles published between 2016 and 2020, searched using electronic databases such as Cochrane library, PubMed, Medline (Ovid), SCOPUS, RISS, and Korea Med, by using Endnote X8.0.1 software (Clarivate Analytics, Philadelphia, PA, USA). The search terms followed as MeSH term: “simulation,” “nursing education,” and “nursing,” and “students,” combined. The search strategy and data extraction are illustrated by the PRISMA check list.

### 2.3. Inclusion Criteria

Inclusion criteria were (1) studies published in English (2) studies related to simulation-based learning, (3) studies focused evaluation strategies to find the outcomes of the simulation-based learning, (4) study participants are nursing students, (5) intervention or comparative study or mixed methods, and (5) availability of free full text by electronic searches or using an institutional site.

### 2.4. Exclusion Criteria

The exclusion criteria of this review were (1) non-English studies, (2) studies are abstract presentation or reviews or clinical trial or conference or cross-sectional studies, and (3) studies are related non nursing education or combined with other health profession.

### 2.5. Data Evaluation and Reduction

Data extraction the articles were properly selected, and data were extracted by using a form that included: author’s name, publication year, methods, instruments used, and main results. As done in the selection process, the extracted data were reviewed by two researchers to ensure the data reliability. In total, 1330 articles were examined.

### 2.6. Data Synthesis

The article synthesis revealed four themes based on the measurement variables for determining the effects of SBL: Knows A Self Com_p, such as knowledge and skills; Attitude; Self (learning, efficacy, determination, competency, confidence, utilization, satisfaction, and assessment); and Com(*n*) (competency, communication, and confidence) and P (perceptions and performance). In addition, this review considered most recently published articles and focused on simulation education conducted by Korean nursing students for the purpose of developing simulation-based learning design in detail for further study.

## 3. Results

**Figure 1:**
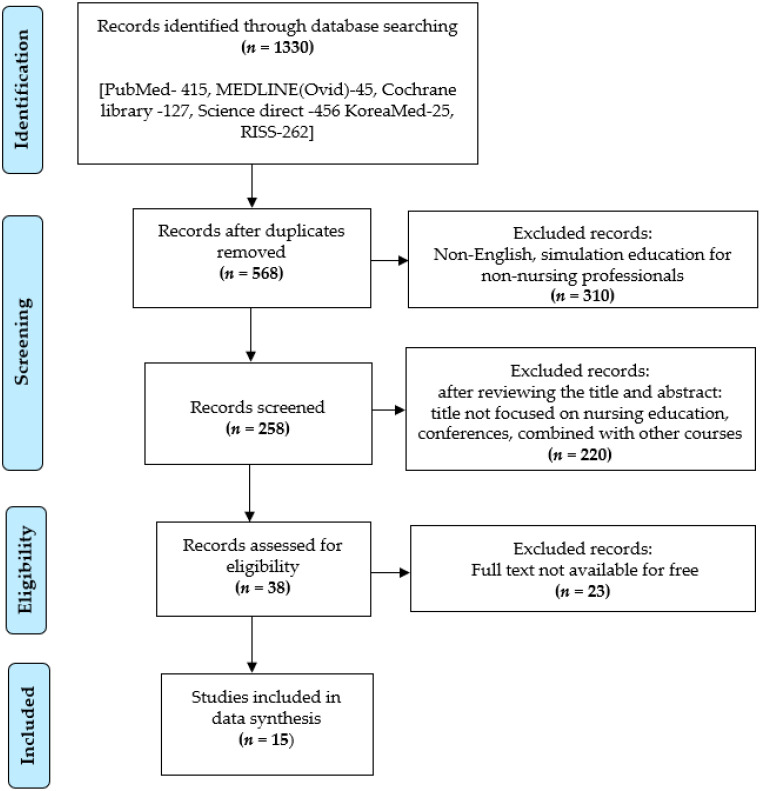
Flow chart for the literature search and article selection process.

The electronic search identified 1330 articles based on the database source. There were 568 articles retrieved after the removal of duplications. After screening, 310 articles were excluded which did not meet the inclusion criteria and 258 articles were selected for the screening. In the screening stage, 220 records were extracted after the titles and abstracts were carefully read due to the presence of only abstracts; these extracted records included reviews, conference papers and simulation education for nursing students combined with other health or short term courses such as technical courses, volunteer programs, disaster management courses, etc. In the eligibility step, 38 articles were considered for in-depth review; however, 23 articles were excluded due the non-availability of the entire text. A total of 15 articles were finalized for the data synthesis (Figure 1).

The review articles focused on the development of scenarios for various health conditions and settings, measurement items, and key findings. The synthesized data revealed that nursing students indicated acquired knowledge, skills, self-efficacy, performance and competency and self-satisfaction as a result of the simulation. Conflicting information was noted regarding the simulation fidelity and the use of scenarios with or without mannequin equipment in simulation-based learning, as a number of simulators lack such equipment, which can create a difference in the meaningful learning experiences obtained.

### Articles’ Characteristics

**Figure 2:**
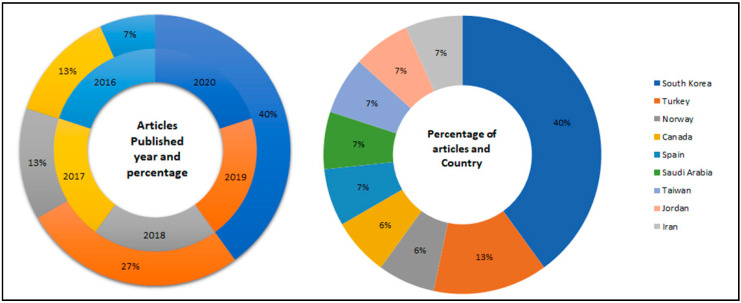
Percentage of articles according to the published year and country of publication.

Out of the 15 articles selected for the final synthesis, the most frequent publication year was 2020 *(n* = 6), followed by 2019 *(n* = 4), 2018 *(n* = 2), 2017 *(n* = 2), and 2016 *(n* = 1). Six articles were published in South Korea, two in Turkey, and one article each in Jordan, Iran, Saudi Arabia, Canada, Spain, Taiwan, and Norway (Table 1, Figure 2).

The main objectives of the articles were to examine the effectiveness of SBL and its competence or performance among nursing students in various settings such as a classroom, clinical areas, nursing units, simulation rooms, and home environments. All study participants were nursing students, most of them seniors or 4th grade students *(n* = 490), and were oriented towards client care in clinical practice. Most participants were in the second grade *(n* = 504), followed by all grades *(n* = 371), juniors *(n* = 120), the third and fourth grades *(n* = 100), and the third grade *(n* = 54).

Most of the articles reviewed were experimental studies *(n* = 10) with intervention or SBL or experiments or tests, or education groups and control groups [11,12,14,15,17,18,19,22,24,25]. There were three mixed method studies [11,20,23], one semi-experimental study [13], one evaluation study [21], and one study based on the Q-methodology [16].

The simulation process and procedure varied according to each study’s objectives. Most of the simulation studies focused on patient care, such as palliative care [11], acute care [18], fall prevention [22], COPD care [24], care of patients with a deteriorating condition [14], and standardized patient care [16]. Simulations relating to techniques covered CPR [23], computer-based [20], and skills training [13]. Some students used simulators in a simulation room with demonstration and reality-based learning; students were divided into groups, such as those learning with low- and high-fidelity simulators [17], branching path simulation [21], and evaluation of respiratory sounds [25]. Theory oriented simulations were lecture material, audio visual aids, and video teaching material distributed to the students after which the effects of SBL were assessed. The descriptions of the articles are presented in Table 1 and the assessment of the effects of SBL is summarized in Table 2.

## 4. Discussion

### 4.1. Knowledge and Skills

Knowledge and skills were measured to evaluate various types of teaching and learning methods and their effects. The review results revealed themes derived from SBL effectiveness. The concepts of knowledge and skills are always interrelated to meeting achievement challenges because nurse educators often focus on developing both knowledge and skills simultaneously while possessing an attitude suitable for traditional educational systems. In this review, knowledge and skills are assessed using various tools. In a previous study [12], students showed improvement in SBL with LBL compared to the control group. The review of SBL provides evidence that simulations are useful in creating a learning environment that contributes to knowledge, skills, safety, and confidence. A tool to impart knowledge about the management of falls was developed by Kim CG [26] and supported by Shin et al. [27]. Its use indicated that simulation-based education demonstrated medium to large effects and could guide nurse educators with regard to the conditions under which patient simulation is more effective than traditional learning methods.

### 4.2. Attitude

Attitude refers to someone’s opinions or feelings about something (e.g., proud behavior or body language). In this study, nursing students participated in many SBL activities and expressed their own feelings and opinions towards the SBL program. A previous study developed a Situation-Background-Assessment-Recommendation (SBAR) fall simulation program using a randomized control pretest post-test design for third-year nursing students in the SBAR group *(n* = 26) [22]. The control group *(n* = 28) focused on the fall simulation program in three stages of scenario development. The study results revealed that the SBAR group showed improvement in all variables compared to the control group.

### 4.3. Self-Realization Based Effectiveness

Seven studies used self-related assessment before and after SBL experiences such as self-directed learning [12], self-efficacy [13,17,20] and self-assessment [13]. Post-scenario self-assessment showed higher competence than pre-scenario self-assessment (*p* < 0.001). Other parameters assessed were: self-confidence [21,23,25] and self-satisfaction [11,15,21,23,25].

Self-directed learning involves the conceptualization, design, conduct, and evaluation of a learning project that is directed by the learner. Ko and Kim [12] conducted a study on SBL among senior nursing students divided into two groups: a group that received simulation-based education (SBE group team-based learning (LBL) (*n* = 86) and an SBL group *(n* = 98). Effectiveness of the simulation scenario was assessed using the Group Readiness Assurance Test (GRAT) and Individual Readiness Assessment Test (IRAT). The SBE with LBL group showed more improvement compared to the SBE group.

Self-efficacy reflects confidence in the ability to exert control over one’s own motivation, behavior, and social environment. Mohamed and Fashafsheh’s [17] used in their study of simulation training using low-and high-fidelity simulators with third-and fourth-grade nursing students *(n* = 100) assessed students’ communication skills and self-efficacy based on their competence. All study participants showed significantly positive self-efficacy and communication skills (<0.001). Ukur et al. [13] noted in their study titled “Simulation based skills training among first-year nursing students *(n* = 65) in theory and skills training,” that post-scenario results revealed high self-efficacy (*p* < 0.05).

Self-confidence refers to a feeling of trust in one’s abilities, qualities, and judgment. A Q-methodology study by Ha focused on nursing students’ experience with standardized patient care in SBL at nursing units among 4th grade nursing students *(n* = 47); SPs improved nursing students’ confidence and nursing competency. This was very helpful for patient care and demonstrated the efficacy of SBL. Masha’al. [21] also reported that perceptions of nursing students about the design utilization of simulation-based Branching path simulation (BPS) improved self-confidence. In addition, Demirtas et al. [23] reported that cardiopulmonary resuscitation (CPR) training focused on SBL for fourth grade nursing students *(n* = 89) resulted in a high level of self-confidence to handle emergency situations post-learning.

Self-assessment is an individual review performed to identify elements that can be improved or exploited to achieve certain predefined goals. SBL methods could be considered in relation to self-assessment in educational intervention outcome variables [28]. Haukedal et al. [14] examined this in an experimental study with second-grade nursing students divided into a control group *(n* = 69) and an intervention group *(n* = 68). The scenario focused on SBL of the First2Act Model of a patient who developed a deteriorating condition. The results revealed that the experimental group scored highly on theoretical knowledge and confidence in implementing the intervention. Post-scenario self-assessment showed higher competence than that in a pre-scenario (*p* < 0.001). In addition, effect sizes were higher in studies of simulation-based evaluation than in those using self-assessment, examinations, or grades for evaluation. SBL increased scores on knowledge and skill examinations [28,29,30].

### 4.4. Communication, Competence and Confidence

The communication ability successfully or accurately supports one’s performance during interaction, which is vital for effective practice in nursing care. For assessing communication skills, it is necessary for a variety of approaches to be used. Ko and Kim [12] assessed the communication skills of nursing students in their study that used SBL for six weeks with three sessions. Participants were senior nursing students who were directly eligible for client care in clinical practice. The study used the GRAT and IRAT for trial evaluation of communication skills. The results showed that the SBE group with team-based education had better communication skills than the control group. Choi et al. [20] conducted a study on computer simulation education with nursing students *(n* = 131) using classroom-focused interactive communication. The education group was given the compEd software education program, whereas the control group had a desktop or tablet PC. The education group showed greater improvement in interactive communication than the control group.

Competence and confidence are based on the possession of sufficient knowledge or skills in a specific area of specialization. Some articles focused on nursing students’ competence in SBL based on the theory and practice of nursing specialties. Ukur et al. [13] reported that simulation-based skills training among first-year nursing students showed that post-scenario results were more strongly associated with students’ competency and confidence than pre-simulation training. Another study focused on Q-methodology of standardized patient care showed the same results and improvement in competence among nursing students [16]. Hung et al. [18] explained that, for simulation learning of acute care for adults, the test group perceived greater competence than the control group. The effectiveness of simulation as a method of competency assessment showed improvement in self-competency, team competency, and performance competency in clinical settings. 

### 4.5. Performance and Perceptions

Performance is a process of carrying out or accomplishing an action, task, or function. In our study, four articles focused on nursing students’ performance of pre-and post-intervention with SBL. An experimental study by Jang et al. with a test group and control group and a total of 226 nursing students assessed the students’ performance regarding an oncology nursing simulation program. Haukedal et al. [14] focused on SBL for treating a patient who developed a deteriorating condition [13], also supported by another study while simulation-based skills training and effectiveness of simulation-based education [12].

Perception is a way of regarding, understanding, or interpreting something: a mental impression. Two articles focused on perceptions and perceived skills regarding SBL. A study explored perceptions towards using branching path simulation (BPS) as an effective interactive learning method, with nursing students *(n* = 52) at a nursing school, for pain management in people with dementia. Results revealed that nursing students’ perceptions were strongly positive toward BPS [21] and recommended implementation of SBL in conditions related to care in old age. Hung et al. [18] reported that the test group of nursing students in a course on acute care for adults found that SBL was very helpful, and the test group of nursing students demonstrated greater competence than the comparison group.

## 5. Conclusions

This review focused on the effects of SBL in nursing education and methods of evaluation to predict its effectiveness. There were numerous studies on health-related SBL for health professions. It is postulated that the studies analyzed present low scientific evidence, indicating the need for further in-depth studies on a topic. This study concludes that SBL as a teaching-learning method is used worldwide as an alternative to the traditional teaching method. However, it is still limited to clinical oriented aspects in undergraduate nursing courses. Many of the studies used the experimental design, typically comparing the simulation and control groups. Some of the articles focused on theory-oriented simulation studies, which indicate that SBL needs to be associated with other strategies and encourage the exploration of all the specialties of the nursing curriculum. The overall results indicate that SBL is a positive, safe, and effective method for nursing students in clinical and simulation room settings to improve the skills and practice of client care directly or indirectly.

### Applying Research to Practice

Nursing students need to educate those who have duties in nursing care. To promote education quality, it is important for nursing students to educate others using high quality education methods. Providing SBL entails positive improvements of the skills and practices used for client care. This research could be the guide for nursing students regarding SBL.

## 6. Limitations

Our integrative review of existing studies regarding the effects of SBL among nursing students from published scientific articles, but our study did have limitations. It is possible that we did not find all of the articles on this subject, due to variance in key words or search terms. In addition, the widely varying SBL education methods in the studies may limit the generalizability of the review’s findings. However, it is true that SBL is a positive method for nursing students’ education, meaning integrative reviews will need to be conducted again.

## Figures and Tables

**Table 1:** Description of the articles included for synthesis.

No	Author/Year/Country	Purpose	Setting and Population	Design	Simulation Process	Assessment of Effects	Outcome
1	Sarabia-Cobo CM et al., 2016 Spain [11]	To evaluate a learning intervention in palliative care using a low-fidelity clinical simulation	Clinical area	Mixed descriptive study	Low fidelity simulation on palliative care	Knowledge Belief Satisfaction	Training for home care was helpful and realistic
			Senior nursing students SBE and TBL (*n* = 86) SBE group *(n* = 98)				
					Scenario 1: Care of terminal cancer patient at hospital Scenario 2: Care of cancer patient at home		
2	Ko & Kim, 2017 South Korea [12]	To identify the effects of simulation-based education combined with team-based learning (SBE combined with TBL) compared to simulation-based education (SBE)	College of nursing	Quasi-experimental study Non-equivalent control group pretest-posttest study	Effectiveness of Simulation	Self-directed learning Communication skills, Performance Confidence Team efficacy, and Team performance	Greater improvement shown by the SBE combined TBL group than by the SBE group
			Senior nursing students SBE & TBL (*n* = 86) SBE group *(n* = 98)		Duration - 6 weeks (3 h/week and 3 sessions) Sessions I: IRAT and GRAT assessment II: Trail and evaluation III: simulation training and debriefing		
3	Ukur K U et al., 2017 Turkey [13]	To evaluate the effects of simulation-based learning on the self-efficacy and performance of first-year nursing students To examine students’ pre- and post-scenario proficiency	College of nursing	Semi-experimental study	Simulation based skills training	Self-efficacy Self-assessment competence Communication Establish Patient safety Safe transfer patient Body mechanism	Post-scenario self-assessment showed higher competence than during the pre-scenario assessment
			1st year nursing students (*n* = 65)		1. Theory class (12 h) - Proper communication with the patient (4 h), - Safe patient transfer (2 h) - Body mechanism (2 h). 2. Skills training (20 h).		
4	Haukedal et al., 2018 Norway [14]	To assess f theoretical knowledge before and after implementation of the intervention r	College of nursing	Quasi-experimental study	First2Act Model	Core knowledge, Assessment, simulation, Reflective review Performance and Feedback	The intervention group had significantly higher scores than the control group *p* < 0.001.
			2nd grade nursing students Control group (*n* = 69) Intervention group (*n* = 69)		- Six scenarios: the patient developed a deteriorating conditions (Angina pectoris, cardiac arrest, hypoglycemia, postoperative bleeding, worsening of obstructive lung disease, and ileus)		
5	Jang et al., 2019 South Korea [15]	To evaluate the effects of an oncology nursing simulation program	Classroom	Non-equivalent control group pretest-posttest design	Oncology nursing simulation program	Knowledge Nursing performance ability Educational satisfaction	The experimental group showed higher performance ability
			Junior nursing students Exp (*n* = 25) Control *(n* = 29)		Themes (management of) Pain, safety, side effects of chemotherapy and emergency situations Experimental group: A lecture (2 h) simulation program (6 h) Control group Case-based learning (6 h), and lecture (2 h).		
6	Ha EH 2018 South Korea [16]	To examine nursing students’ experience with standardized patients in simulation-based learning	Nursing Unit	Q-methodology	Standardized patient care	Helpful for patient care (patient-centered view), Important for learning (SPs roles-centered view), Promotion of competency	SPs improved nursing students’ confidence and nursing competency
			Fourth grade nursing students (*n* = 47)		Steps 1 Q Population, a Process of Creating the Q Sample Step 2 Q Sample (Q set), a Process of Developing Q Sample Statements Step 3 Sample (P set), a Process of Recruiting Study Participants Step 4 Q Sorting, a Process of Ranking Q Samples Sorted to three domains (strongly agree, +4; neutral, 0; strongly disagree, −4) Step 5 Factor Extraction (Data Analysis) scores ranging from 1 to 9, scores were inputted into the pc-QUANL program		
7	Mohamed and Fashafsheh 2019 Saudi Arabia [17]	To evaluate the effects of simulation-based training on nursing students’ communication skill, self-efficacy and clinical competence in practice	Classroom	Quasi experimental design	Simulation training on low and high-fidelity simulators.	Communication Skill Self-Efficacy Clinical Competence	Simulation-based training, showed improvement
			3rd and 4th grade Nursing students (*n* = 100)		Duration: 6 Weeks (6 h/week) Sections 1. Orientation and pre briefing 2. Education with demonstration and video teaching (10 min) 3. Re-demonstration Debriefing and feedback		
8	Hung CC et al., 2019 Taiwan [18]	To explore the effects of simulation-based learning (SBL) on nursing student c competencies and performance in the clinical setting	Simulation center	Comparison group study	Acute care for adults’ course	Competency Professional knowledge Technical skills; Nursing process; Communication; Critical thinking	Test group perceived greater competences than comparison group
			2nd grade nursing students test group (*n* = 49) Control (*n* = 51)		Duration: 10 teams and 1.5 h Phases 1: introduction, II: scenario display, and III: debriefing		
9	Harder N et al., 2019 Canada [19]	Development and highlighting of the implementation of the simulation activities	Clinical setting	Experimental study	Manikin and non manikin-based simulation	Prioritization of Skills	Absence of a mannequin lowered the skills of the students.
			Nursing students (*n* = 120)		Scenario focused on 5 patients with varying needs Chart and prioritize needs (15 min) Meet and conduct evaluation (20 min) Group discussion and debriefing (15 min)		
10	Choi et al.,2020 South Korea [20]	To compare the efficacy of a computer simulation-based interactive communication education	Classroom	Mixed method study	Computer simulation education	Communication Knowledge Learning Self-efficacy Communication- efficacy	The intervention group showed improvement than the attention group
			Nursing students EG *(n* = 66) ACG *(n* = 65)		Education group ComEd program installed on a tablet PC Attention group Tablet PC or desktop PC All students were asked to complete the questionnaire thrice (before, the, immediately after and two weeks after the program)		
11	Masha’al 2020 Jordan [21]	To explore perceptions towards using BPS as an effective interactive learning method	Nursing School	Evaluative study	Branching path simulation (BPS)	Perceptions Design, Utilization Self-confidence Satisfaction	Perception was strongly positive towards BPS.
			Nursing students (*n* = 52)		Scenario: pain management in people with dementia		
12	Jeong and Kim 2020 South Korea [22]	To develop a Situation-Background-Assessment-Recommendation (SBAR) fall simulation program	College of Nursing	Randomized control pretest posttest design	Fall simulation program	Knowledge & Skills Attitude Communication ability Communication clarity	SBAR group showed more improvement in all variables than the control group
			3rd grade nursing students SBAR group *(n* = 26) Control *(n* = 28)		Stage 1 (45 min) Formal introduction Distribution of lecture material, handouts related to falls Stage 2: Role play (60 min) stage 3: Summary (15 min)		
13	Demirtas A et al., 2020 Turkey [23]	To examine the effectiveness of a simulation-based CPR training program on the knowledge, practices, satisfaction, and self-confidence	Emergency Unit	Mixed method	Cardiopulmonary resuscitation (CPR) training	Knowledge Skills Self-confidence and Satisfaction	Simulation found to be an interesting and useful teaching method with a high level of self-confidence.
			*4th grade nursing students* *(n = 89)*		Pretest Distribution of assessment tool Simulation 10 students in each team (15 min each students) Summary and debriefing (10 min) Posttest Assessment of self-satisfaction		
14	Torkshavand G et al., 2020 Iran [24]	To determine the effects of simulation-based learning on students’ skills in providing education to older patients	Clinical area	Quasi-experimentalstudy	Care of patients with COPD	Knowledge and skills (all *p*’s < 0.001).	Improvement in knowledge and skills in the SBL group than the LBL group
			4th grade nursing students SBL *(n* = 35) LBL *(n* = 35)		Session 1: Pre-briefing Session 2: Patient education module team (20–25 mons each students) Session 3: Debriefing			
15	Üzen Cura et al., 2020 South Korea [25]	To compare the effect of different simulation modalities on knowledge, skill, stress, satisfaction, and self-confidence	Clinical area	Experimental study	Evaluation of Respiratory Sounds	Knowledge Skill Stress level Student satisfaction Self-Confidence	Post practice knowledge levels of the three groups showed similar knowledge. Reduced stress and increased satisfaction and self confidence
			2nd grade nursing students (*n* = 266) From 3 nursing schools		Team (8–10 students) Three different simulation modalities: standardized patient, high-fidelity mannequin, and partial task trainer Assessment (10–13 min)		

SBL, simulation-based learning group; LBL, lecture-based learning group; GRAT, group readiness assurance test (GRAT); IRAT: Individual Readiness Assessment Test. EG, Education group; ACG, Attention control group.

**Table 2:** Themes of simulation-based learning (SBL) in nursing education.

Themes	Assessment of Effect	References
Knows	Knowledge and skills	Sarabia-Cobo CM et al. (2016) [11], Haukedal et al. (2018) [14], Jang et al. (2019) [15], Hung et al. (2019) [18], Choi et al. (2020) [20], Jeong & Kim (2020) [22], Demirtas A et al. (2020) [23], Torkshavand G et al. (2020) [24], Üzen Cura et al. (2020) [25],
A	Attitude	Jeong & Kim (2020) [22]
Self	Self-directed learning Self-efficacy Self-assessment Self-confidence Self-satisfaction	Ko & Kim (2017) [12], Ukur K U et al. (2017) [13], Ha EH (2018) [16], Mohamed &. Fashafsheh (2019) [17], Choi et al. (2020) [20], Demirtas A et al. (2020) [23], Üzen Cura et al. (2020) [25]
Com_P (Competence)	Competency	Mohamed &. Fashafsheh (2019) [17], Hung et al. (2019) [18], Ha EH (2018) [16]
	Confidence	Ko & Kim (2017) [12], Demirtas A et al. (2020), Cura S U et al. (2020)
	Communication	Ko & Kim (2017) [12], Ukur K U et al. (2017) [13], Mohamed &. Fashafsheh (2019) [17], Hung et al. (2019) [18], Choi et al. (2020) [20], Jeong & Kim (2020) [22]
	Performance	Ha EH (2018) [16], Haukedal et al. (2018) [14], Jang et al. (2019) [15]
	Perceptions	Masha’al (2020) [21]

## Data Availability

Data sharing not applicable.
